# Cost-Effectiveness and Budget Impact Analysis of Apatinib for Advanced Metastatic Gastric Cancer from the Perspective of Health Insurance System

**DOI:** 10.1155/2017/2816737

**Published:** 2017-11-26

**Authors:** Yongrui Bai, Yuejuan Xu, Bin Wu

**Affiliations:** ^1^Department of Radiation Oncology, Ren Ji Hospital, School of Medicine, Shanghai Jiao Tong University, Shanghai, China; ^2^Department of Clinical Oncology, The Second Hospital of Nanjing Affiliated with the Medical School of South East University, Zhongfu Road 1, Nanjing, China; ^3^Department of Pharmacy, Ren Ji Hospital, School of Medicine, Shanghai Jiao Tong University, Shanghai, China

## Abstract

**Objective:**

This study evaluated the cost-effectiveness of apatinib in patients with chemotherapy-refractory mGC.

**Patients and Methods:**

A Markov model was developed to simulate the clinical course of typical patients with chemotherapy-refractory metastatic gastric cancer (mGC). We estimated the 10-year quality-adjusted life-years (QALY), costs, and incremental cost-effectiveness ratios (ICER). Model inputs were derived from the published literature and government sources. Direct costs were estimated from the perspective of the Chinese health insurance system. A scenario analysis for a Patient Assistance Programme (PAP) was performed.

**Results:**

Baseline analysis showed that apatinib increased the cost and QALYs by $7859 and 0.192, respectively, relative to conventional chemotherapy, resulting in an ICER of $40,997/QALY gained. When PAP was available, the ICER was $21,132/QALY. Probabilistic sensitivity analyses confirmed that apatinib with PAP achieved nearly 65% likelihood of cost-effectiveness at the threshold of $22,200. One-way sensitivity analyses demonstrated that the utility of progression-free survival was the most influential factor on the robustness of the model. Budget impact analysis estimated that the annual increase in fiscal expenditures would be approximately 0.45 million dollars.

**Conclusions:**

Our analysis suggests that apatinib is likely cost-effective in patients with chemotherapy-refractory mGC when PAP is available.

## 1. Introduction

Gastric cancer is the fourth most prevalent malignant cancer worldwide, and more than 700,000 people die from the disease annually [1]. Over two-thirds of new cases and deaths occur in developing countries, and 42.4% occur in eastern Asia. The incidence rates per 10,000 Chinese males and females were 28.7 and 13.8, respectively [2]. Although early detection is becoming more common, the majority of patients have locally advanced or metastatic disease at the time of diagnosis. Systemic chemotherapy based on the combination of fluoropyrimidine and platinum is widely accepted as a palliative treatment that substantially improves overall survival and quality of life compared with single-agent chemotherapy or best supportive care [3]. However, the prognosis for advanced gastric cancer remains poor, especially in metastatic gastric cancer (mGC) patients who fail second-line chemotherapy [4]. Clearly, the poor overall survival (OS) of advanced gastric cancer indicates that new treatments with acceptable toxicity profiles are urgently needed.

VEGF has been described as the most important, potent angiogenic factor linked with growth and metastatic spread of several tumour types. The VEGF family includes different isoforms, VEGF-A, VEGF-B, VEGF-C, and VEGF-D, as well as placental growth factor (PlGF). VEGF-A binds to VEGFR1 and VEGFR2, VEGF-B and PlGF bind to VEGFR1, and VEGF-C and VEGF-D bind to VEGFR-2 and VEGFR-3. High VEGF concentrations have been related to vascular dissemination and poor outcomes in patients affected by GC [5]. Therefore, as a novel target, blocking VEGFR-2 could be a promising strategy to inhibit tumour-induced angiogenesis. Various VEGFR-2 inhibitors, including receptor-specific antibodies, and low molecular weight chemicals, such as sorafenib, vandetanib, cediranib, and sunitinib, have recently been developed.

Apatinib, also known as YN968D1, is one of the most recent oral antiangiogenic agents with encouraging preclinical and clinical data in the treatment of a variety of solid tumours. It could inhibit VEGF-stimulated endothelial cell migration and proliferation and decrease tumour microvascular density [6]. Several basic studies have been dedicated to evaluating the antitumour activity of apatinib in vitro and in vivo [7]. A phase 1 clinical study showed that a partial response was noted in seven patients (18.9%) and stable disease in 24 (64.9%), and there was a disease control rate (DCR) of 83.8% at 8 weeks among 37 evaluable patients [8]. In patients who experienced treatment failure with at least two chemotherapeutic regimens, phases 2 and 3 also demonstrated that apatinib treatment has statistically significant differences between the apatinib arms and placebo arm for the progression-free survival (PFS) and OS (*P* < 0.001) [9, 10]. The safety profile was acceptable, and the regimen was well tolerated. Evidence suggests that apatinib confers clinical benefits for patients with chemotherapy-refractory mGC; One latest cost-effectiveness analysis from the perspective of Chinese society showed that apatinib is not a cost-effective option [11]. However, the financial implications of apatinib as a third-line treatment have not been examined to date.

The objective of the present study was to evaluate the cost-effectiveness of apatinib treatment as a third-line treatment for patients with chemotherapy-refractory mGC. A Chinese health insurance perspective was adopted to help determine the direct economic value of apatinib.

## 2. Methods

### 2.1. Decision Model Structure

**Figure 1:**
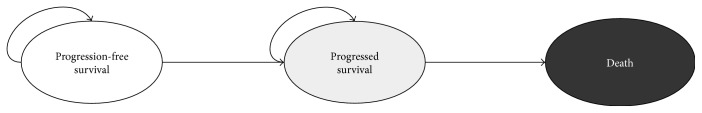
Simplified model structure based on the Markov process illustrating the two strategies for treating metastatic gastric cancer.

A mathematical model based on the Markov process was developed to evaluate the ten-year clinical and economic outcomes associated with metastatic gastric cancer (mGC) and its treatment, which consisted of 3 mutually exclusive health states: progression-free survival, progressed survival, and death. The structure of the model is presented in Figure 1. In the Markov model, the cycle length is one week due to the short life expectancy, and the entry state is progression-free survival. During each Markov cycle, the patient either remained in his or her assigned health state or progressed to a new health state. R statistical environment (version 3.2.2; R Development Core Team, Vienna, Austria) was used for model construction and data analysis.

A hypothetical cohort that was clinically similar to those patients with mGC in the phase III trial of apatinib was entered into the model [9, 10]. The hypothetical patients were 18–70 years of age with histologically confirmed advanced gastric cancer or mGC (including gastroesophageal junction adenocarcinoma) for which they had experienced treatment failure with at least two chemotherapeutic regimens. They would receive 1 of 2 competing strategies to manage mGC (third-line therapy): (1) supportive care (control strategy) or (2) 850 mg of apatinib per day (apatinib strategy). After cancer progressed, patients were all assumed to be managed with supportive care based on the Chinese oncologists' opinion.

The primary outcomes were disease-free life-years (LYs), overall LYs, quality-adjusted life-years (QALYs), and cost. The cost and QALYs were discounted at an annual rate of 5%, which is in line with the Chinese guidelines for pharmacoeconomic evaluations [12, 13]. The costs are shown in 2015 US dollars. Incremental cost-effectiveness ratios (ICERs), which indicate the cost per additional QALY gained, were examined. The current analysis used $22,200 per QALY gained (3 × per capita GDP of China in 2015) as the cost-effectiveness threshold according to the World Health Organization (WHO) recommendations [14–16].

### 2.2. Clinical Data

**Figure 2:**
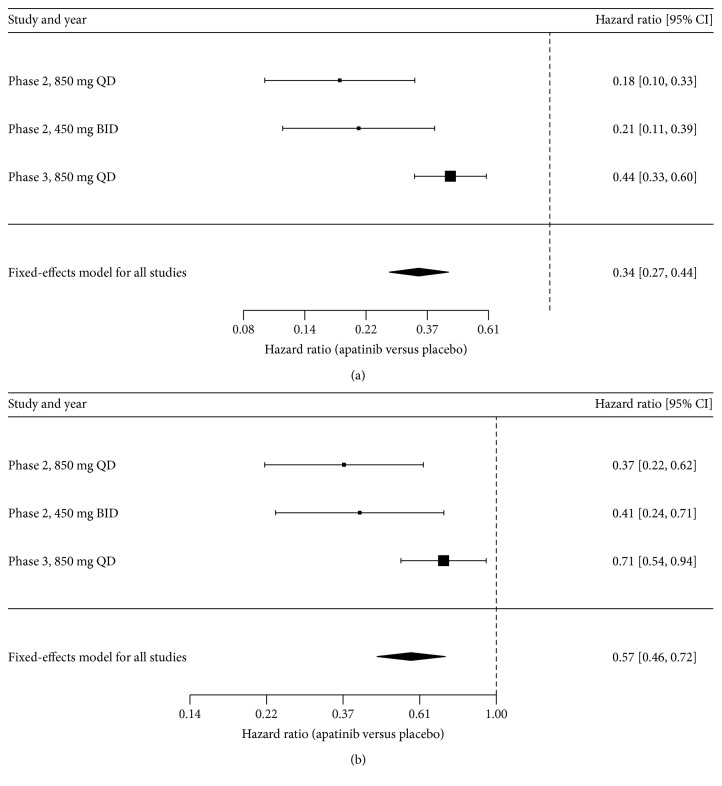
Forest plot of the meta-analysis for the PFS (a) and OS (b).

Transition parameters and proportions (Table 1) were derived from randomized clinical trials. Kaplan–Meier survival curves for the progression-free survival (PFS) and overall survival (OS) for the control strategy were available in phase 2 and phase 3 clinical trials. Weibull curves were fitted to the data extracted from the Kaplan–Meier curves using R statistical software. The estimated scale and shape parameters, standard errors (SEs), adjusted *R*^2^, and correlation coefficients are described in Table 1. The shape parameter (*γ*) allows the hazard function to increase or decrease with increasing time; for *γ* > 1.0, the hazard rate strictly increases in a nonlinear pattern with increasing time. The scale parameter (*λ*) is related to the measurement unit of time. The HR of the PFS and OS for apatinib versus placebo was estimated by meta-analysis as shown in Figure 2, which included the phase 2 and 3 trials of apatinib treatment for patients with mGC who do not respond to or who experience progression with second-line chemotherapy [9, 10]. The reference survival rates at each cycle were calculated and weighted according to the patient numbers. [17] Once a survival rate was calculated, the survival rates for the active strategies were adjusted with the following formula: *S*_active strategies_ = *S*_IFN‐*α*(reference)_^HR^ (HR: hazard ratio).

### 2.3. Medical Costs and Utility

The costs of each strategy (Table 2) were estimated from the perspective of Chinese health insurance, and the direct medical costs are considered in the model. The critical illness insurance would cover 60% of the medical expenditures [18–20].

The estimated treatment costs were based on the following schedule: 850 mg of apatinib would be administered daily until disease progression and supportive care would be prescribed for patients with disease progression. The cost of supportive care was derived from a previously published report. Due to the similar frequency of severe adverse events (SAEs) between the apatinib and control placebo arms [9, 10], resource use associated with treatment-related SAEs was not included in the current analysis.

Because of the high price of apatinib, affordability of apatinib in China is weak. The apatinib Patient Assistance Programme (PAP) was implemented for Chinese patients with mGC. Patients paid for the first three months of apatinib and then received free medication in the subsequent months until disease progression (3 + X PAP). Therefore, the impact of PAP was evaluated in the scenario analyses.

Utility values for progression-free and progressive disease were derived from the reported study [21] and are described in Table 3. The utility of the progression-free survival was 0.88 and was used for both strategies, as there was no difference in the quality of life when apatinib was added to chemotherapy because the adverse drug events were not significantly different between the two strategies. For the state of progressed survival, a utility of 0.41 was assigned.

### 2.4. Sensitivity Analyses

To explore the model robustness, one-way and probabilistic sensitivity analyses (PSA) were performed. In the PSA, key model parameters were simultaneously and randomly sampled from the set parametric distributions to produce 1000 estimates of the cost and QALY in each strategy. Triangle distributions were chosen for cost parameters, and the beta distribution was utilized for the probability, proportion, and preference value parameters. A cost-effectiveness acceptability curve (CEAC) would be presented based on the PSA results. One-way sensitivity analyses were performed for all parameters at a priori defined ranges shown in Tables 1 and 2, which were mainly obtained from previous studies or assuming 25% or 50% of the base-case value.

### 2.5. Budget Impact Analysis

A Markov-based budget impact model was performed to estimate the direct medical expenditures for patients with mGC based on a Chinese governmental perspective over 5 fiscal years. The model measured the direct medical costs of two treatments as the primary outcome in this analysis. We specifically focused on the incremental cost of treatment when adding apatinib to the coverage list. The incidence of GC was nearly 21.55 per 1,000,000 people [2], including 2/3 with advanced disease. Based on expert opinion, we assumed that apatinib with 3 + X PAP would be available for eligible patients from the pharmaceutical company and 60% of the 3-month medication cost would be subsidized by the government.

## 3. Results

### 3.1. Base-Case Analyses

Our model projected the costs and health outcomes of two strategies with or without PAP. The apatinib strategy produced a greater increase in the QALYs over the course of the disease (0.458 compared to 0.267 QALYs for the control strategy), which can largely be explained by the PFS associated with each strategy. Compared to the control strategy, the marginal costs of apatinib were $7859 and $4051 without PAP, resulting in ICERs of $40,997 and $21,132, respectively (Table 3).

### 3.2. Sensitivity Analysis

**Figure 3:**
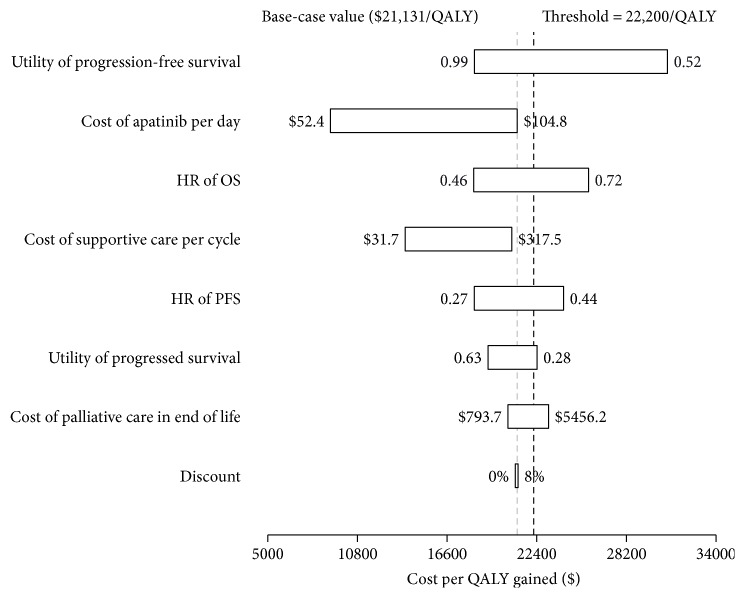
A tornado diagram representing the one-way sensitivity analysis of apatinib with 3 + X PAP versus the control strategy. OS: overall survival; PFS: progression-free survival; HR: hazard ratio.

The ICERs of apatinib with 3 + X PAP versus the control strategy were plotted for a range of values of the model inputs. The most sensitive variables are shown in the tornado diagrams (Figure 3). The results suggested that the most sensitive parameters were the utility of progression-free survival, cost of apatinib, HR of the OS and PFS, and proportion of the fee paid by insurance. Other parameters, such as the cost of supportive care, had medium or little impact on the model outcome.

**Figure 4:**
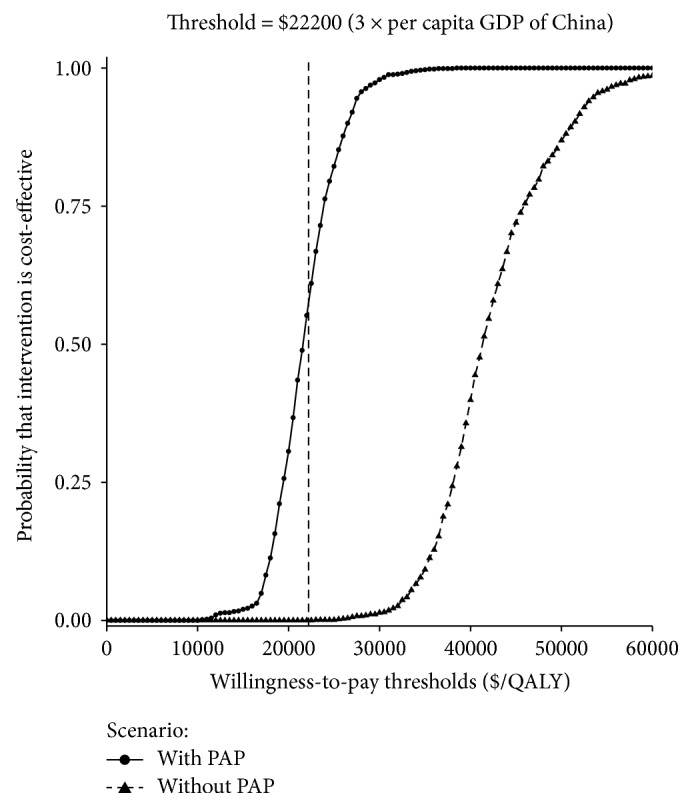
The cost-effectiveness acceptability curves for apatinib strategies with or without PAP compared to the control strategy. The *y*-axis indicates the probability that a strategy is cost-effective across the WTP per QALY gained threshold (*x*-axis). The bold vertical dashed line represents the threshold for China.

The PSA results are shown via cost-effectiveness acceptability curves (Figure 4). The proportions of simulations that were cost-effective for apatinib treatment without PAP and with 3 + X PAP were approximately 0% and 65%, respectively.

### 3.3. Budget Impact Analysis

**Figure 5:**
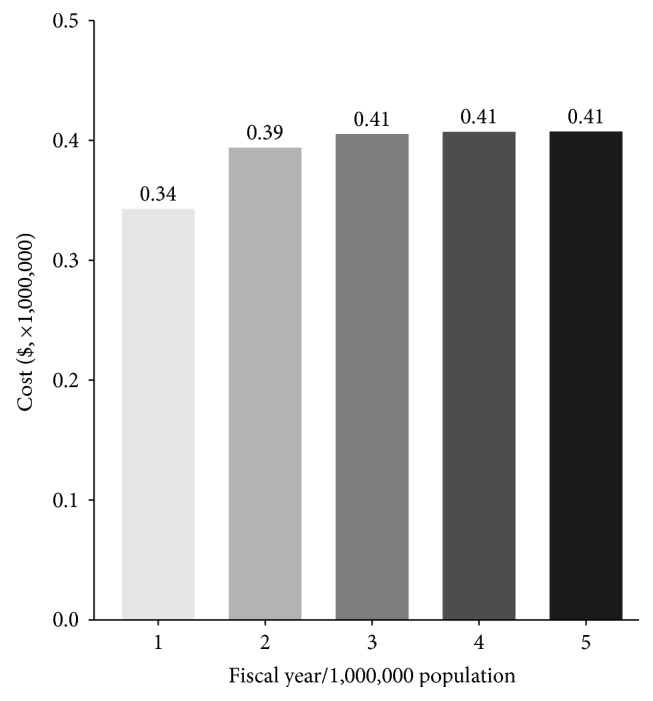
Estimated budget impact during fiscal years 2016 to 2020 with the provision of apatinib per one million population.

The budget impact analysis estimated how the prescription of apatinib to patients with mGC would impact future expenditure if the health insurance system covers apatinib in the Medicare plan. The estimated number of patients with indications for trastuzumab in gastric cancer is approximately 215 cases per million population. The budget impact on the government health fiscal burden from 2016 to 2020 is presented in Figure 5. In the first years, the incremental costs would be approximately 0.34 million dollars. Following the second fiscal year, the annual increase in fiscal expenditures would be constant at nearly 0.41 million dollars.

## 4. Discussion

When new treatment options for advanced cancer become available, it is necessary to evaluate their health economic impact before they are accepted by health insurance systems, especially in resource-limited settings such as China. Gastric cancer has a heavy societal burden for China. Apatinib treatment improves the survival for mGC patients. However, it has a high cost. Economic evaluation of apatinib for treating mGC is under increasing scrutiny. Using a Markov analytic model, we found that the 10-year ICER for third-line therapy based on apatinib was generally unfavourable, with a value of $40,997 per QALY gained. When 3 + X PAP of apatinib was available, the ICER was decreased to $21,132/QALY, indicating that apatinib with PAP is a cost-effective alternative in China. Probabilistic sensitivity analysis suggested that this result was robust. Many Chinese local governments have established a catastrophic disease insurance system [19, 20], and providing apatinib with 3 + X PAP in a hypothetical area with a population of one million would annually consume approximately 0.41 million dollars based on the budget impact analysis. The current analysis also showed that apatinib was not a cost-effective option when no PAP was available, which was inherent with the previous study [11]. However, our analysis found that the ICER of apatinib over control strategy was $40,997/QALY, which was lower than the published result ($90,154.00/QALY) [11]. The following is the potential reasons: (1) our analysis only included the costs paid by health insurance; (2) Exponential and Weibull survival distributions were used for estimating the transition probabilities in their analysis and our analysis, respectively. However, exponential survival parametric model can only be used when the hazard is a constant [22]; (3) one month and one week were chosen as Markov cycle in their analysis and our analysis, respectively. However, a shorter cycle length could improve the accuracy of the model output [23].

The potential ability of apatinib, the first targeted agent for chemotherapy-refractory mGC, to improve survival was a major determinant of the clinical and economic outcomes. A one-way sensitivity analysis showed that the HRs of the OS and PFS for apatinib versus placebo were two of the most influential parameters based on the model robustness. When the HR of the OS or PFS was adjusted to the lower and upper values, the ICER of apatinib with 3 + X PAP versus the control strategy became more favourable and exceeded the threshold of $22,200/QALY. This result indicates that the choice of the patient subgroup could increase the cost-effectiveness of apatinib treatment. Target gene screening for medical decision making with targeted agents could improve the health economic outcomes [24]. A challenge to the use of apatinib is the need to find biomarkers that can predict drug efficacy. A study of biomarkers for apatinib in breast cancer patients showed that both hypertension and high expression of p-VEGFR2 could be biomarkers for good treatment efficacy [25]. However, there are no standardized, established biomarkers for apatinib. If such biomarkers were identified in clinical studies, apatinib treatment for patients with those biomarkers would have more favourable outcomes. Other independent, influential parameters include the utility of progression-free survival and price of apatinib. Due to the high quality of life in the PFS healthy state, a favourable PFS with apatinib could increase the QALYs. An additional scenario analysis showed that apatinib treatment with 2 + X or 1 + X PAP in patients with mGC would lead to more favourable ICERs. A special discount plan for Chinese health insurance might be helpful.

Several important limitations in the current economic analysis should be considered. For example, we did not fully explore other potential therapies for treating chemotherapy-refractory mGC due to the absence of clinical evidence. Second, the sensitivity analysis indicated that the HR of the OS and PFS had an important impact on the economic outcomes. In the current analysis, the model extrapolated survival beyond the trial follow-up. Therefore, the greatest model uncertainty might be attributed to the long-term survival rates. However, because of the low survival probabilities of chemotherapy-refractory mGC at 2 years and the well-matched calibrated curves of survival from the Weibull model, we are confident that no significant bias was introduced in the current analysis. Third, the current model did not consider the impact of adverse events because apatinib is a relatively clean tyrosine kinase inhibitor such that most adverse reactions would be somewhat alleviated after treatment is stopped. Nevertheless, we are confident that the model accurately represents the common clinical conditions of chemotherapy-refractory mGC in China. We hope that this paper will be an important reference for Chinese decision makers who are evaluating whether to approve apatinib coverage.

## 5. Conclusion

Our analysis indicates that apatinib therapy with 3 + X PAP in patients with chemotherapy-refractory mGC would be cost-effective in China. It could be helpful to include apatinib coverage in health insurance.

## Figures and Tables

**Table 1:** Clinical data.

Parameters	Values	Description and reference
Weibull survival model of the control arm PFS	Scale = 0.04191; shape = 1.4165; *r*^2^ = 0.972	[9]
Weibull survival model of the control arm OS	Scale = 0.02143; shape = 1.18716; *r*^2^ = 0.989	[9]
HR of PFS (apatinib versus control)	0.34 (95% CI: 0.27–0.595)	[9, 10]
HR of OS (apatinib versus control)	0.57 (96% CI: 0.537–0.937)	[9, 10]

**Table 2:** Base-case costs estimates ($, year 2013 values) and utilities.

Parameter	Median	Range	Description and reference
Costs			
Cost of 425 mg of apatinib	106.5	53.2~106.5	Local charge
Cost of palliative care in end of life	1483.9	1072.3~2119.3	Calculation
Cost of supportive care per cycle	117.1	32.3~322.6	Calculation
Utilities^&^			
Utility of disease-free	0.88	0.8~0.97	Measured
Utility of recurrent disease	0.41	0.28~0.63	Measured

^&^The values were measured by the time trade-off (TTO).

**Table 3:** Summary of the cost and outcome results in base-case analysis.

Strategy	Control (US $)	Apatinib (no PAP)	Apatinib (3 + X)
Cost in disease-free state	529	4215	7868
Cost in disease recurrence state	902	1224	1306
Cost in death for gastric cancer	1033	1076	1149
Total cost ($)	2464	10,323	6515
Disease-free LYs	0.173	0.360	0.360
Overall LYs	0.471	0.750	0.750
QALYs	0.267	0.458	0.458
Incremental cost per LY^∗^ (US $)		28,170	14,520
Incremental cost per QALY^∗^ (US $)		40,997	21,132

^∗^Compared with the control arm.
